# Effect of head and limb orientation on trunk muscle activation during abdominal hollowing in chronic low back pain

**DOI:** 10.1186/1471-2474-15-52

**Published:** 2014-02-22

**Authors:** Kevin Parfrey, Sean GT Gibbons, Eric J Drinkwater, David G Behm

**Affiliations:** 1 School of Human Kinetics and Recreation, Memorial University of Newfoundland, St. John’s, Newfoundland, Canada; 2 Faculty of Medicine, Memorial University of Newfoundland, St. John’s, Newfoundland, Canada; 3 School of Exercise and Health Sciences, Edit Cowan University, Perth, Australia

**Keywords:** Chronic low back pain, Exercise therapy, Motor control, Abdominal hollowing, Primitive reflexes, Stability exercise

## Abstract

**Background:**

Individuals with chronic low back pain (CLBP) have altered activations patterns of the anterior trunk musculature when performing the abdominal hollowing manœuvre (attempt to pull umbilicus inward and upward towards the spine). There is a subgroup of individuals with CLBP who have high neurocognitive and sensory motor deficits with associated primitive reflexes (PR). The objective of the study was to determine if orienting the head and extremities to positions, which mimic PR patterns would alter anterior trunk musculature activation during the hollowing manoeuvre.

**Methods:**

This study compared surface electromyography (EMG) of bilateral rectus abdominis (RA), external oblique (EO), and internal obliques (IO) of 11 individuals with CLBP and evident PR to 9 healthy controls during the hollowing manoeuvre in seven positions of the upper quarter.

**Results:**

Using magnitude based inferences it was likely (>75%) that controls had a higher ratio of left IO:RA activation with supine (cervical neutral), asymmetrical tonic neck reflex (ATNR) left and right, right cervical rotation and cervical extension positions. A higher ratio of right IO:RA was detected in the cervical neutral and ATNR left position for the control group. The CLBP group were more likely to show higher activation of the left RA in the cervical neutral, ATNR left and right, right cervical rotation and cervical flexion positions as well as in the cervical neutral and cervical flexion position for the right RA.

**Conclusions:**

Individuals with CLBP and PR manifested altered activation patterns during the hollowing maneuver compared to healthy controls and that altering cervical and upper extremity position can diminish the group differences. Altered cervical and limb positions can change the activation levels of the IO and EO in both groups.

## Background

Over 80% of chronic low back pain (CLBP) occurrences are of unknown origin [1]. Many different treatment protocols have been used by physiotherapists and other clinicians [2]. One of the proposed reasons for the development of CLBP is an altered pattern of neuromuscular control of the spine [3-9]. There are numerous differences in the motor system when CLBP subjects are compared to normal subjects [10]. One finding is that the deep muscle system is impaired, while the superficial muscles are variable with some displaying increased activity [9].

One treatment protocol for CLBP has been performing abdominal hollowing [4,11-14]. During a standardized exercise for abdominal hollowing, the activation of the larger superficial muscles such as the rectus abdominis (RA) are increased with CLBP patients [4]. The RA and external oblique (EO) muscles are global muscles responsible for gross movements of the trunk. The RA is the major trunk flexor while the EO are more responsible for lateral flexion and rotation [15]. It is theorized that the global muscles are substituting for the decreased amount of force, which the stabilizing muscles no longer supply [4,6]. It is thought that if a specific exercise program is administered, which revolves around retraining the proper activation patterns during abdominal hollowing, that the altered pattern and function can be corrected [4]. The goal of this intervention is not to increase the strength of the abdominals, but rather to retrain the altered motor pattern of the abdominal musculature associated with CLBP.

While this type of treatment has been shown to be effective in treating CLBP patients by increasing function and decreasing pain levels [4], some patients have difficulty learning how to perform the hollowing maneuver. O’Sullivan et al. [16] reported that some individuals with CLBP took 4–5 weeks to properly learn and perform the hollowing maneuver, while Gibbons [17,18] has identified a subgroup who cannot learn specific exercise and has recommended sub-classifying this group. This group is characterized by self reported neurocognitive, sensory motor, gross motor and respiratory deficits, as well as physical findings of sensory deficits and neurological soft signs (i.e. primitive reflexes : PR) [19]. Neurological soft signs are deviations in motor, sensory and integrative functions that do not signify localized brain dysfunction [20]. The presence of PR such as tonic neck and Moro reflexes within a CLBP population may indicate a deficiency in the supraspinal control of the anterior trunk muscles [17-19]. This may contribute to altered patterns of superficial muscle activity during abdominal hollowing [19].

PR are brain stem mediated complex automatic movement patterns which are evoked through touch or changes in body position [21,22]. The disappearance of PR are a sign of central nervous system (CNS) development as it indicates cortical inhibition, which is necessary for voluntary movement [23]. The presence of PR in adults have been associated with neurological disorders such as Alzheimer’s and dementia [24,25]. It is also theorized that the recurrence of PR in adults may be an inherent consequence of usual aging [25]. As CLBP has been associated with atrophy of CNS gray matter, and specifically GABAergic inhibitory interneurons [26], then PR may resurface due to a decrease level of inhibition on the brainstem neurons responsible for the autonomic movement patterns. It may be possible that CLBP lead to alterations in the CNS and that the presence of PR is an indication of this change. Likewise this presence may explain why some individuals with CLBP have difficulty learning how to perform the hollowing maneuver. However, research is scant on this topic and more research is necessary to substantiate this possibility.

The objectives of this study were to examine if (1) there was a difference between the abdominal activation patterns of a CLBP group with apparent PR and a matched healthy control group when performing the hollowing maneuver; (2) by altering the position of the head and limbs to mimic that of a PR the CLBP group would have a similar activation pattern to the control; (3) there is a side specific activation pattern of the superficial abdominal muscles in either the CLBP group or the control group.

## Methods

### Subjects

Twenty participants (9 control and 11 CLBP) completed the experiment. Participants were split into a CLBP with prevalent PR group (Height: 163.6 ± 9.1 cm, Weight: 79.6 ± 19 kg, Age: 45.6 ± 9.9 years) or a control group without a history of CLBP (Height: 163.3 ± 9.9 cm, Weight: 78.8 ± 15.3 kg, Age: 42.3 ± 8 years). The control group was age, gender and mass matched to eliminate differences associated with different demographics and morphology. All subjects were explained the procedures of the study, given an opportunity to ask questions for clarification and made aware that they could stop the study at any point. All subjects were required to read and sign a consent form before participation. The Memorial University Human Investigation Committee approved the study (#09.184).

Inclusion for the CLBP group was identified by a score of over 12 on the Rolland Morris Disability Questionnaire (RMDQ) [27] and suffering from low back pain for greater than 12 weeks [1]. Subjects were excluded from the CLBP group if there was a presence of severe postural abnormality and/or signs and symptoms of specific LBP including: radicular symptoms, radiological diagnosis (specifically spondylolisthesis or spondylolysis); limited neck range of motion or pain; known factors associated with primitive reflexes (severe postural abnormality, anti-depressant medication, opiate medication diabetes, previous neurological incidents, neurological conditions, heart surgery, diagnosed learning difficulties, withdrawal from alcohol or drug addictions or psychiatric conditions). A certified physiotherapist assessed the presence of PR. Intra-tester reliabilty of PR assessment has previously been established [28]. Exclusion criteria for the control group were any report of low back pain in the previous 6 months [29], limited neck range of motion or neck pain, or if they had any conditions (same as above) known to be associated with the presence of retained PR.

A novel approach was that an inclusion criterion for the CLBP group was the presence of at least one PR. PR presence was based on a 0–4 rating scale from absent to the full pattern present [30]. If no remnant of the reflex was found, the rating was zero. If any aspect of the reflex pattern was present, it was deemed to be present and was rated then from 1–4 with 4 being the full reflex pattern. For assessment of the asymmetric tonic neck reflex (ATNR), the individual was placed in the supine position with the upper limb by the side. The ATNR was deemed present if active cervical rotation was accompanied by ipsilateral shoulder girdle elevation and/or the contralateral leg appears to shorten. For the secondary assessment of the ATNR, the individual stand with feet shoulder width apart and shoulders flexed to 90°. The ATNR was deemed present if active cervical rotation was followed by a weight shift to the side of rotation or ipsilateral shoulder girdle movement. For assessment of the stage 1 phase of the Moro reflex the individual was placed in the supine position with the glenohumeral joint in 30° abduction and 90° elbow flexion. The reflex was deemed present if active cervical extension to 30° was followed by lumbar spine extension, hip extension or glenohumeral external rotation. Phase 2 of the Moro reflex was performed in supine crook lying. It was deemed present if active cervical flexion to 30° was accompanied with posterior pelvic tilt, hip adduction or flexion, glenohumeral internal rotation or elbow flexion.

### Procedures

#### Electromyography

**Figure 1:**
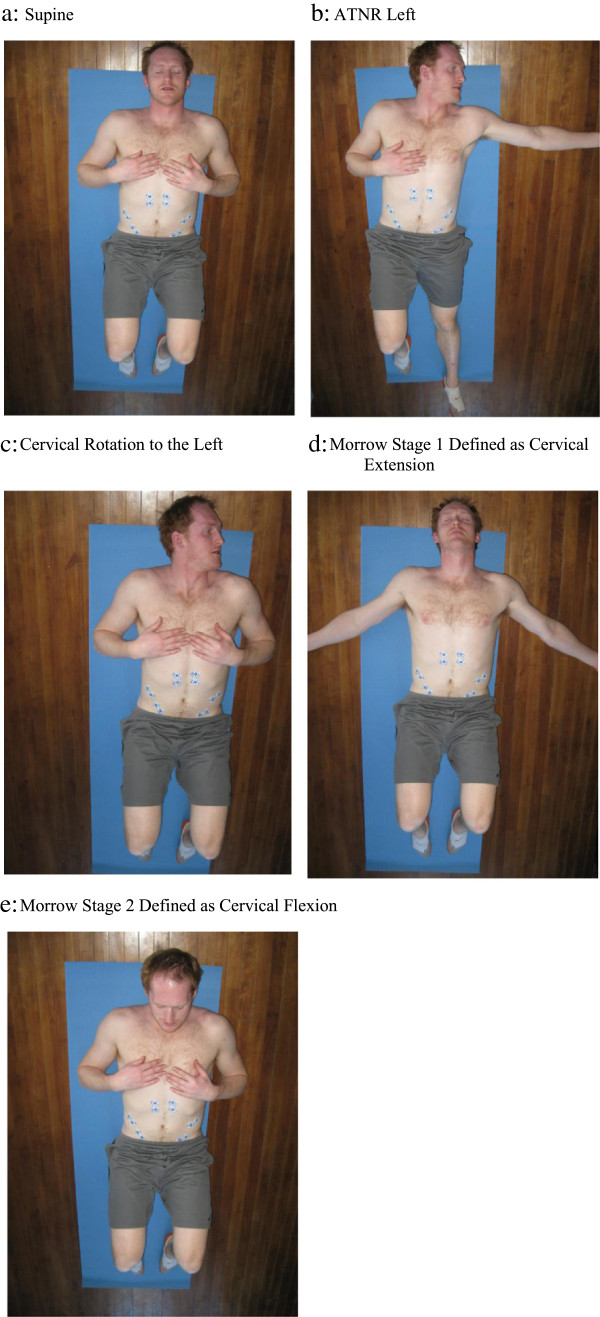
**Electrode placement and exercise position.** Acronyms: ATNR: Asymptomatic Tonic Neck Reflex. Consent was obtained from the researcher in the photos. **a**: Supine, **b**: ATNR Left, **c**: Cervical Rotation to the Left, **d**: Morrow Stage 1 Defined as Cervical Extension, **e**: Morrow Stage 2 Defined as Cervical Flexion.

The subjects were instructed to lie flat on a horizontal bench and were fitted bilaterally with surface electrodes on the RA, EO, and internal obliques (IO). All surface electrodes (Meditrace 130 ECG Conductive Adhesive Ag/AgCl Electrodes, Tyco Healthcare Group LP, Mansfield, MA) were placed bilaterally on six different abdominal muscle sites. To reduce resistance of the signal, all sites for electrode placement were shaved, scrubbed with sand paper and rubbed with an alcohol-soaked paper towel [31-35]. This process removed body hair, dead skin cells, and oils [31-35]. Based on previously published articles from this laboratory, all electrodes were placed parallel to the muscle fibres, with an interelectrode difference of 2 cm [31-35]. The bilateral sites were the RA, which was defined as five centimetres below the xiphoid process and three centimetres lateral to the midline. The EO electrodes were placed five cm superior to the anterior superior iliac spine (ASIS) while the IO were placed immediately medial to the ASIS (Figure 1). All the described EMG sites have been used in a number of previous studies published from this laboratory [31-35]. The surface electrode site identified as IO may also detect EMG activity from the transversus abdominus (TrA) muscles as well [23-27]. A ground electrode for each of the six sites was placed over the iliac crest.

All EMG signals were collected over a 20 second period, sampled at 2000 Hz with a Blackman -61 band-pass filter between 10–500 Hz, and amplified (500X) (Biopac Systems MEC bi-polar differential 100 amplifier, Santa Barbara CA., input impedance = 2 M, common mode rejection ratio > 110db min (50/60Hz), noise > 5 UV). EMG activity was then directed through a 12 bit analog-to-digital converter (Biopac MP 100) and stored on a computer. Based on the successful procedures from previous studies [34,35], EMG activity was analyzed over a 3 second period corresponding to the change in the biofeedback pressure monitor from 40–50 mmHg. Each site at each position had two successful trials, which were rectified and integrated, these two trials were averaged. The average integral of each muscle and exercise was normalized to the rectified integral of the same muscle during the double straight leg raise test. Raw EMG signals were visually inspected for issues such as saturation and low signal to noise ratios. These data were removed from analysis. The researcher was blinded as to which files were controls or CLBP.

After the EMG electrode set up was complete, the subject was asked to perform a double leg raise exercise, which would be used for normalization of the data. After the normalization procedure was completed, the subject was instructed on how to successfully perform the hollowing maneuver in a supine crook lying position.

#### Double leg raise exercise

Subjects were asked to lie supine on a bench with their hips flexed to 45°. On the investigators mark the subject would raise both feet 1 cm off a plinth and hold the position for ten seconds. This exercise was then used to normalize the exercise EMG data. A submaximal isometric contraction was performed for normalization since maximal contractions are known to be unreliable in a CLBP population [36]. The double leg raise exercise was selected because it has been shown to activate all the abdominal muscles of interest to stabilize the pelvis during the maneuver [4]. Further, a maximal contraction may cause an aggravation of symptoms in those with CLBP and since this protocol was used previously [4], it allows a more consistent comparison of the studies.

#### Abdominal hollowing maneuver

The abdominal hollowing maneuver as previously described [4,11-14] was performed by the subjects. Subjects would lie crook lying with a Pressure Biofeedback Unit^TM^ (PBU) (ProTherapy Supplies, Duluth Georgia) placed under the lordotic curve of the spine between S1 and L1 (i.e. the lumbar spine was in a neutral position) to ensure the subject was able to control for anterior pelvic tilt. The PBU was set to 40 mmHg. The test-retest reliability of the the PBU has been previously reported as 0.81 [37]. Subjects were asked to perform abdominal hollowing. They were instructed to do this through several verbal cues. The head and trunk were to remain stationary and subjects were told to not flatten their back, flex forward or push through their arms or feet. They were also told to keep breathing and not hold their breath. When the subject could successfully complete the hollowing maneuver with a slow and gradual onset and hold it for ten seconds three different times the experiment continued.

Pushing through their feet, posterior pelvic tilting or flexing the trunk are strategies in which the PBU can be changed without properly performing the abdominal hollowing maneuver, therefore these were considered an unsuccessful performance. It was determined if the subjects pushed through their feet by placing weight scales (My Weigh Scales^TM^, CanadianWeigh, Toronto, Ontario) under their feet. Breathing was monitored visually and by a capnograph (CapnoTrainer®, Better Physiology Ltd. Santa Fe, New Mexico). Trunk movement was monitored by monitoring the ASIS as well as gross and quick changes to the PBU. The abdominal hollowing was performed in a manner that would gradually bias transversus abdominis, then bias IO (while still hollowing the abdominal wall). When the pattern of execution was satisfactory as determined by the physiotherapist, the subject was then asked to perform the hollowing maneuver until they were able to change the pressure in the PBU at a steady state from 40 to 50 mmHg. The subjects would then hold this isometric activation and keep the pressure at 50 mmHg for at least ten seconds in order to control for anterior pelvic tilt.

Next the subject would perform the hollowing maneuver in six different randomized body positions three times each for ten seconds. If the investigator noticed any problems in the execution of either the double leg raise exercise or the hollowing maneuver the subject was asked to stop given a break of thirty seconds and asked to retry the exercise for one additional repetition. Electromyographic (EMG) data were taken throughout all of the exercises. When the experimenter saw that the participants had changed the pressure from 40 to 50 mmHg it was marked as the starting point to which EMG would be analyzed for comparison. The first three seconds of successful performance were used unless, it was noticed by the experimenter that there was a pressure change at some point in the ten second activation, in which case it was noted that a different starting point should be used for the three second EMG analysis.

### Simulated primitive reflexes

The six supine body positions (Figure 1) used in the experiment were positions that attempted to mimic the orientation of the body if a specific PR was stimulated. A position similar to the ATNR was chosen because it was the most frequently observed retained PR in infants with neurological lesions [22] and adult with CLBP [28]. As well, it has the potential to influence abdominal hollowing if present. Rotating the head at least 15° in either direction stimulates the ATNR. The reflex causes the limbs to which the head is pointing to extend and the contralateral limbs to flex [22]. Two positions used in this study were cervical rotation to either the left (Figure 1b) or right with the arm (side to which head is pointed) extended straight out and perpendicular to the torso and the leg (side to which head is pointed) extended. The arm on the opposite side to which the head pointed was flexed with the hand laid on the chest and the leg of the same side was flexed 45° at both the hip and knee. Another two positions were simple cervical rotation to the left (Figure 1c) or right with their arms crossed on their chest and legs/hips flexed at 45°.

Another position incorporated in this study attempted to simulate the Moro reflex. This reflex is stimulated by cervical extension in the supine position and has two stages [38]. Stage one occurs immediately after cervical extension, which elicits extension and abduction of the upper extremities (Figure 1d), stage two is the return to a fetal position and involves cervical flexion (Figure 1e) along with upper extremity flexion and adduction [38]. Both stages of the cervical and upper limb aspects of the Moro were mimicked in this study. Stage one was simulated by having the subject extend at the cervical spine as far as possible without causing pain and arms abducted to approximately 60° resting on the plinth (Figure 1d). Stage two was simulated by having the subject flex at the cervical spine as far as possible without causing pain as well as having the arms resting on the subject’s chest (Figure 1e). When the end point of cervical flexion was achieved a triangular pad was placed under the head so it could rest at that position. If end range flexion exceeded that of the pad the subject was asked to bring their head back until it was resting on the pad, which was placed to hold the maximum amount of flexion. Hips and knees were flexed to 45° for both stages.

### Statistical analysis

To avoid the shortcomings of research based in null-hypothesis significance testing, magnitude-based inferences and precision of estimation were employed [39]. Magnitude-based inferences on the clinical (practical) difference in abdominal muscle activation patterns between a CLBP group and a matched control in different body positions when performing the hollowing manoeuvre.

Precision of estimated (mean) differences between the control and CLBP groups were calculated using unpaired t-tests on log-transformed data, then back-transformed and expressed with 95% confidence limits to define the range representing the uncertainty in the true value of the (unknown) population mean. Qualitative descriptors of standardized (Cohen) effect sizes were calculated as the difference between means divided by the standard deviation of the control (supine) condition and assessed using these criteria: trivial < 0.2, small 0.2-0.5, moderate 0.5-0.8, large >0.8 [40]. Effect sizes were also calculated on the lower and upper 95% confidence limits so the mean and variability of all dependent variables could be compared on a common metric. Effects with 95% confidence limits substantially overlapping the thresholds for small positive and negative effects (exceeding 0.2 of the standard deviation on both sides of the null) were defined as unclear. Clear small or larger effect sizes (i.e. those with > 75% likelihood of having an effect size of at least small, as calculated by a previously available spreadsheet [41] were defined as substantial [42]. This analysis was performed on the normalized data as well as a ratio of IO:RA. The IO:RA ratio has been shown to be the best representation of hollowing maneuver performance in previous research [4,16] as the goal of the exercise is to emphasize activation of IO and TrA while minimizing activity of RA.

## Results

### Between group differences for IO:RA ratio

**Figure 2:**
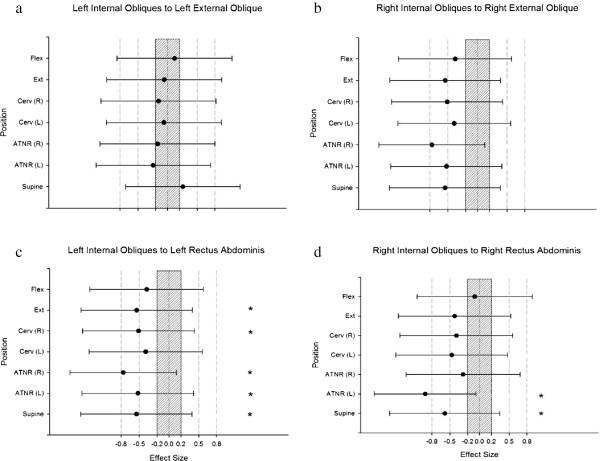
**Figures plot standardized effect size differences between control and chronic lower back pain groups when comparing a) left internal obliques to left external obliques, b) right internal obliques to right external obliques, c) left internal obliques to left rectus abdominus, and d) right internal obliques to right rectus abdominus.** Each graph represents a muscle group with plots representing the magnitude of difference between ratios between muscle groups between the two groups in the different postures. Positive values indicate the chronic lower back pain group had higher normalized values. Error bars indicate 95% confidence limits of the mean difference between groups. The shaded area of the graph indicates the region in which the difference between groups is trivial (i.e. between -0.20 and 0.20 standardized effect sizes). Asterisks (*) indicate conditions with >75% likelihood that the difference exceeds the smallest worthwhile difference.

Using magnitude based inferences, for the left IO:RA ratio the control group would be at least 75% more likely to have a substantially greater ratio in the supine (cervical neutral position), ATNR left and right, cervical rotation to the right and cervical extension positions (d = -0.54, -0.52, -0.77, -0.51 and -0.54 respectively; all “moderate”) than the CLBP group. Similarly on the contralateral side it was at least 75% more likely that the control group would have a greater right IO:RA ratio than the CLBP group in the supine (cervical neutral) and ATNR left positions (d = -0.58 and -0.91, respectively “moderate” and “large”). A greater IO:RA ratio represents relatively less RA EMG activity (Figure 2).

### Between group differences for normalized site specific activation levels

**Figure 3:**
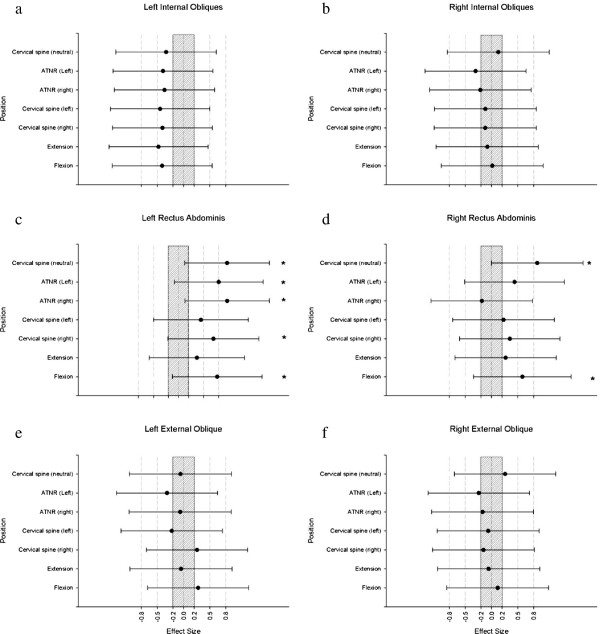
**Graphs plot standardized effect size differences between control and chronic lower back pain groups for a) left internal obliques, b) right internal obliques, c) left rectus abdominus, d) right rectus abdominus, e) left external obliques, f) right external obliques.** Each graph represents a muscle group with plots representing the magnitude of difference between the two groups in the different postures. Positive values indicate the chronic lower back pain group had higher normalized values. Error bars indicate 95% confidence limits of the mean difference between groups. The shaded area of the graph indicates the region in which the difference between groups is trivial (i.e. between -0.20 and 0.20 standardized effect sizes). Asterisks (*) indicate conditions with >75% likelihood that the difference exceeds the smallest worthwhile difference.

Analysis of confidence limits and effect sizes illustrated <75% likelihood of a clinical difference between the two groups in any position for the IO or EO sites (Figure 3). There were however, likely clinical differences between groups for the RA. For the left RA it was likely that the CLBP group would have greater activation than the control in the supine (cervical neutral) (d = 0.97, “large”), ATNR left (d = 0.80, “large”) and right (d = 0.97, “large”), cervical rotation to the right (d = 0.70, “moderate”) and flexion (d = 0.77, “moderate”) positions (Figure 3). For the right RA it was likely that the CLBP group would have greater activation than the control in the supine (cervical neutral) (d = 0.87, “large”) and cervical flexion position (d = 0.59, “moderate”) (Figure 3).

Statistical significance can be inferred from the 95% confidence limits. If 95% confidence limits cross the zero, the mean must have a p > 0.05, because the lower limit is less than zero while the upper limit is greater than zero. If, however, the 95% confidence limits in the figures are both on the same side of the zero, the mean has a p < 0.05 (Figures 2 and 3).

## Discussion

The results of this paper suggest that during the performance of the hollowing maneuver the CLBP group demonstrated “clinically meaningful” greater RA muscle activation levels (lower IO:RA ratios) compared to matched control groups indicating a bias toward RA when performing an abdominal hollowing exercise. As mentioned above, note that the term “clinically meaningful” indicates the observed difference is of sufficient magnitude and consistency to have at least 75% likelihood of having a substantial (meaningful) impact in a clinical setting. This indicates a spinal or supraspinal response to cervical orientation altered the activation patterns of the anterior trunk musculature.

As far as the authors are aware, this is the first study to assess the influence of altered cervical and limb position on the hollowing maneuver. These positions were intended to mimic the positions of PR. The novel finding in the present study was that activation levels were substantially affected by altered body position. This implies that in certain body positions CLBP patients illustrated an altered motor pattern when attempting to selectively activate their IO, and in other body positions had a motor pattern that clinically resembled the healthy population.

### Effects of cervical and limb positions on motor patterns

While there are different abdominal musculature activation patterns between CLBP patients and healthy populations [3,4,16,43], it is consistent that there is an alteration in the mechanism of how the central nervous system controls the spine [7,44,45]. One of the manifestations is a variable pattern of muscle substitution [9,44]. There are different activation patterns of the abdominal musculature during activities such as postural tasks [46], gait [47], trunk rotation [48], holding a load [49], orthopaedic tests [50,51] and specific exercises [4]. Alterations in muscle activity are considered to be deficiencies in the coordination and control of the abdominal musculature and may result in a less stable spine during movement [52].

The main objective of the abdominal hollowing maneuver is to bias activation of the TrA and IO while minimizing activity of RA. The present study illustrated that it was clinically likely, compared to controls, for the CLBP group to have higher activation levels of the left RA in the supine (cervical neutral), ATNR left and right, cervical rotation to the right and cervical extension positions while performing the hollowing maneuver. Similarly, the CLBP group had substantially higher levels of the right RA in the cervical neutral and cervical flexion positions. This difference illustrates that when performing the hollowing maneuver in these positions the CLBP group required greater RA activation to change the pressure cuff 10 mmHg. It is thought that muscle substitution occurs because the RA attempts to compensate for the deficient IO in the CLBP group [16]. However, there is no previous research demonstrating that an alteration of body position changes the ratio of IO:RA during the hollowing maneuver.

The substantially increased level of left RA activity seen in the CLBP group was not illustrated on the right RA to the same extent. The CLBP group was clinically likely to have higher levels of left RA activity, compared to the control, in the cervical flexion, cervical rotation to the right, ATNR left/right, and cervical neutral positions. The CLBP group was clinically likely to have higher activation of the right RA, compared to the control, in only the cervical flexion and cervical neutral positions only. There are three possible explanations for this result. 1) There were a predominant number of participants with right-sided pain and thus increased contractions on the left side may have been predominant to help brace or stabilize the area. 2) Only certain PR were assessed therefore there could have been other PR or neurological soft signs (i.e. frontal release signs, clumsiness, motor incoordination, difficulty with motor sequencing) present that were not accounted for. 3) Finally the physiotherapist always stood on the right of the participant during instruction of the AHM, which may have affected the individual’s focus for ipsilateral motor recruitment.

The present study did not show any clinically meaningful differences between the two groups for IO activation. This would suggest that there is not a deficiency in the IO magnitude of the CLBP group compared to the control. It is postulated that to attain the 10 mmHg pressure change of the biofeedback cuff must require higher levels of IO activation. Therefore, there may be a ceiling effect for IO for the hollowing maneuver performed in this study. These results agree with O’Sullivan et al. [4,16] who used a similar protocol. Similarly, O’Sullivan et al. [4,16] did not illustrate a significant difference in activation levels of the IO between a CLBP group and healthy control. However, when a ratio of RA:IO was compared there were significant differences between groups, which indicated an altered motor program.

The CLBP group had similar activation levels of the left RA with the cervical rotation to the left and extension positions. Interestingly, these activation levels were altered by changes of cervical orientation and not the extremities. This indicates that the changes in activation were unlikely due to structural changes in the position of the muscle but more likely at the spinal and/or supraspinal level.

Other researchers have investigated the influence of altering limb position on aspects of motor control in humans. Cervical positions are known to alter the accuracy of upper limb movement in healthy people [53-55] and elbow joint position error in subjects with whiplash associated disorders [56]. The head may be used as a reference for the performance of upper limb movements and the altered proprioception of the neck may introduce error in the mechanism of central control of movement. CLBP subjects also have altered proprioception [57]. It is unknown if the trunk uses the cervical spine in a similar manner to the upper limb, however it could be possible that a similar mechanism is involved in the altered motor patterns observed here. Investigations on humans have found an influence of altering neck position on the motor system. Deutsch et al. [58] reported that head repositioning may affect the strength of the upper limb through the influence of the tonic neck reflexes. LePellec and Maton [59] concluded that the tonic neck reflexes can have a small influence on high force production with elbow flexion.

### CLBP-related inhibition mechanisms

In normal function, neck receptor influences on muscle tone are involved with complex postural responses, which also reflect sensory information from visual, vestibular, proprioceptive and somatic sources [30,60]. These will interact strongly to each other and will likely have variable individual influences. Turning the head to one side accentuates the extensor tone of the limbs on that side, with flexion on the contralateral side [60]. Given the different levels of integration of PR in adults, the tone changes with repositioning of the neck can be variable and complicated.

CLBP may be associated with an overall reduction of CNS inhibition. Baliki et al. [61] theorized that the increased activation of medial pre-frontal cortex (PFC) in individuals with CLBP, compared to controls, might be due to a disruption of the mutual inhibitory interactions. A decrease in inhibition could help explain why motor cortical maps increase in volume with CLBP [62,63]. If CLBP causes a decrease in inhibitory interneurons then the altered activation pattern of the anterior trunk muscles of the CLBP group during the hollowing maneuver compared to the control may be due to over activity of the CNS. In terms of muscle substitution, it is generally thought that the RA increases its activation level to make up for a deficient ability to activate IO [16]. While theoretically and functionally this makes sense, recent research on CLBP and brain morphology and activity poses an alternate explanation. With a decrease in gray matter volume and density it is mainly a loss of inhibition that results [26]. Therefore the increased activation levels of RA may be due to an inability to inhibit this activation when attempting to perform the hollowing maneuver. This explanation would support the results in the present study as our CLBP subjects exhibited substantially higher normalized levels of both left and right RA compared to a matched control in a variety of different positions but had similar levels of IO activation. This indicates that while both groups were able to activate IO to a similar extent, however the control group was substantially better at activating IO while minimizing activity in RA.

### Primitive reflexes with CLBP

While reduced inhibition helps explain muscle substitution, it does not clarify why the present study showed that altering cervical orientation can substantially affect RA activation. Subgroups of subjects with CLBP have been found to have significantly higher levels of PR than other groups [28]. PR are brainstem mediated movement patterns which are inhibited by areas in the frontal lobe [64]. PR typically start to be inhibited at six months [21] and their presence is used to assess CNS integrity [23]. While it is unknown whether the PR in this current population has resurfaced, as it does with normal advanced aging, or if they have been present throughout the subjects’ life it can possibly indicate CNS disruption.

Age is an unlikely reason for the resurfaced PR as the population employed had an average age of 45 and PR re-emergence is usually not seen until the sixth decade [65]. Similarly if PR are resurfacing it would agree with the theories that there is an overall reduction in inhibition associated with reduced grey matter and CLBP [26,61]. If PR are present in an individual with CLBP it may be possible that the altered motor strategy is due to a reduction in supraspinal inhibition.

There was an expectation that the muscle activation patterns of the CLBP group would be more similar to the control during performance of the hollowing maneuver when placed in a position mimicking either ATNR or the supine extension reflexes (e.g. Moro reflex, tonic labyrinthine reflex {TLR}). Placing our CLBP subjects in the ATNR position with altered position of the extremities did not substantially affect performance compared to controls. However, cervical rotation to the left with the hollowing maneuver by CLBP, had activation of left RA similar to controls. Likewise, cervical extension illustrated similar levels of RA activation between groups. How cervical orientation affects trunk muscle activation patterns in this study can only be speculative. In this study, the subjects’ body and limb orientations were not performed passively. They actively placed themselves in these positions. Hence it may be possible that the process of consciously placing the limb in a position resembling the PR is what provided inhibition.

Perhaps by inhibiting this reflex with altered head and limb orientations, it is reopening latent inhibitory synaptic pathways in the frontal lobe. This may in turn access other inhibitory pathways allowing the CLBP patients to activate IO while also inhibiting activation of RA during the hollowing maneuver. Wand et al. [66] also came to the conclusion that widespread disinhibition may be a fundamental issue with CLBP and that treatment paradigms that elicit intracortical inhibition should be explored.

### Limitations

This study poses new insight into both muscular activation patterns of CLBP patients as well as how altering cervical orientation can affect these activation patterns. However, the results must be considered within the limitations of the study. In this study, only surface EMG electrodes were used. At the site of IO, there will be recordings from TrA since it lies directly beneath this point. McGill et al. [67] reported that surface electrodes adequately represent the EMG amplitude of the deep abdominal muscles (i.e. TrA and IO) within a 15% RMS difference. Ng et al. [68] indicated that electrodes placed medial to the ASIS would receive competing signals from the EO and TrA with the IO. Based on these findings, the EMG signals obtained from this abdominal location are described in the present study as the IO, which would be assumed to include EMG information from both the TrA and IO. However this limitation should not affect the interpretation of the results in this study for three reasons. 1) Anatomically it has been shown that the lower fibers of both IO and TrA have similar orientation and attachments [69]. 2) Likewise it has been proposed that they have similar synergistic functions in ipsilateral rotation and sacroiliac joint closure [70]. 3) Finally it has been shown that the hollowing maneuver is performed by the combined activity of IO and TrA [71]. Because of the similarity in function and anatomy, these two muscles have been recorded together with surface electrodes in a number of studies from this laboratory and their EMG activity have been differentiated from other neighbouring muscles such as the RA and EO [34-37]. A further limitation of the study was that CLBP patients without PR were not included in the investigation. In addition, palpation for the presence of activation of TrA may also have been helpful but due to the number of researcher responsibilities during the experiment (e.g. monitoring EMG, PBU, subject’s pushing of feet, performance of hollowing maneuver and others), it was not possible to add this additional measure.

The expression of our results may not be familiar to all readers. We purposefully omitted p-values and discussion of statistical significance. While both p-values and 95% confidence limits can be used to infer statistical significance (see last paragraph of results section), 95% confidence limits are much more information-rich to the clinician. The p-value only represents the probability of the mean response to a treatment not being zero. Concluding that a mean was “unlikely to be zero” is not a clinically useful conclusion because it does not express the variability in responses in clinically meaningful units. However, expressing results using a mean in conjunction with upper and lower confidence limits allows clinicians to easily interpret the likely effects they can expect from an intervention.

### Local and global abdominal hollowing

It is clear from the descriptions used in the literature for laboratory research and clinical trials on abdominal hollowing that there are different versions of the exercise in use [12,72,73]. In general, there are two types of abdominal hollowing. The first aims to bias TrA over IO, EO and RA. This may be considered “local abdominal hollowing”. During the hollowing maneuver described here and by O’Sullivan et al. [4,16], it should be noted that the change in the pressure biofeedback unit from 40 mmHg to 50 mmHg does not occur with TrA alone. This 10 mmHg change requires higher levels of superficial muscle activity. Here, the goal of the exercise is to bias activation of IO while minimizing activity of RA. This may be considered “global abdominal hollowing”. The former exercise is designed for translation control of individual spinal segments, while the latter is designed for movement control [4,16]. It should be noted that the ‘global’ abdominal hollowing may be performed as a progression of the ‘local’ abdominal hollowing or independently of it. Invariably, there will be some element of translation control with the ‘global’ abdominal hollowing even if it is performed independently of the ‘local’ abdominal hollowing. This requires further clarification, but is beyond the scope of this paper. In this study, ‘global’ abdominal hollowing was taught as a progression from ‘local’ abdominal hollowing.

The change in the PBU from 40-50 mmHg may also consist of a contribution from a co-contraction with lumbar multifidus since this is known to co-contract with TrA [74]. Given that the change in the PBU depends on a change in the orientation of the posterior abdominal wall, and this may be at least be partially dependent upon intra-abdominal pressure, it may be permissible that coordination of the whole deep muscle system or cylinder (TrA, diaphragm, pelvic floor, psoas major, deep lumbar multifidus) contributes to this change [72]. Although other studies have looked at abdominal hollowing [75,76], only O’Sullivan et al. [4,16] have looked at ‘global’ abdominal hollowing, used EMG and as a monitoring tool and standardized the amount of abdominal hollowing with a PBU.

## Conclusion

Further research is required to understand the mechanism of altering cervical and limb position on abdominal activity, and the influence of other PR or neurological soft signs on CLBP. Clinically, the re-emergence or continuing presence of PR may influence muscle activation patterns of CLBP patients during the hollowing maneuver. A trained physiotherapist was able to identify the presence of PR symptoms in all the participants of the CLBP group. These results may help with the sub-classification of CLBP patients. This could open up new assessment protocols for CLBP patients in which PR presence should be determined. If there are PR present then it can be possible that there is a decrease level of supraspinal inhibition and that the main goal of treatment should not be abdominal hollowing, but learning to inhibit RA when performing the hollowing maneuver. Alternatively, a treatment protocol of PR inhibition may be required especially if the patients have difficulty learning the hollowing maneuver or minimizing RA activity during the hollowing maneuver [77]. Likewise clinicians may find it easier to teach individuals how to inhibit RA by changing cervical orientation [19].

This is the first study to assess the influence of cervical and limb orientation on the hollowing maneuver. These findings should be interpreted with caution. There is some discrepancy in the literature regarding abdominal hollowing and the terminology of the different versions can get combined. There is clinical evidence to support the use of a rehabilitation program that includes ‘local’ abdominal hollowing in chronic and recurrent low back pain through meta-analysis [7] and articular low back pain through systematic review [78]. Although there is clinical support for ‘global’ abdominal hollowing [16], a systematic review or meta-analysis has not been conducted. This would provide further support for this exercise in the management of CLBP.

## Abbreviations

ASIS: Anterior superior iliac spine; ATNR: Asymptomatic tonic neck reflex; CLBP: Chronic low back pain; CNS: Central nervous system; EMG: Electromyography; EO: External obliques; IO: Internal obliques; L1: First lumbar vertebrae; PBU: Pressure biofeedback unit; PFC: Pre-frontal cortex; PR: Primitive reflexes; RA: Rectus abdominus; S1: First sacral vertebrae; SD: Standard deviation; TLR: Tonic Labyrinthine Reflex; TrA: Transversus abdominus.

## Competing interests

There were no competing or conflicting interests in the completion of this study.

## Authors’ contributions

All authors were involved in the idea conception and study development as well as writing and reviewing the paper. KP and SGibbons were involved in data collection. KP also provided the data analysis. All authors read and approved the final manuscript.

## Authors’ information

Kevin Parfrey: Mr. Parfrey completed his Master of Science (Kinesiology) degree from Memorial University of Newfoundland. He also completed a Master of Physiotherapy degree from Dalhousie University in Halifax, Nova Scotia. He presently works as a physiotherapist for KD Physical Therapies in the Halifax region.

Sean Gibbons: Mr. Gibbons is a registered certified physiotherapist and Director of SMARTERehab owner of Stability Physiotherapy in St. John’s, Newfoundland, Canada. He is completing his PhD in clinical epidemiology at Memorial University of Newfoundland.

Eric Drinkwater: Dr. Drinkwater completed his Master of Physical Education degree from Memorial University of Newfoundland. He collected data for his doctoral degree at the Australian Institute of Sport in Canberra, completing his PhD through Victoria University (Melbourne) in 2006. Dr. Drinkwater presently works with School of Exercise and Health Sciences at Edit Cowan University in Perth Australia.

David Behm: Dr. Behm is the Associate Dean for Graduate Studies and Research in the School of Human Kinetics and Recreation at Memorial University of Newfoundland in St. John’s, Newfoundland, Canada.

## Pre-publication history

The pre-publication history for this paper can be accessed here:

http://www.biomedcentral.com/1471-2474/15/52/prepub
